# An equity and efficiency integrated grid-to-level 2SFCA approach: spatial accessibility of multilevel healthcare

**DOI:** 10.1186/s12939-021-01553-9

**Published:** 2021-10-19

**Authors:** Shaoyao Zhang, Xueqian Song, Jie Zhou

**Affiliations:** 1 Present Address: College of Geography and Resources Science, Sichuan Normal University, Chengdu, 610066 China; 2 School of Management, Chengdu University of Information Technology, Chengdu, 610225 China; 3 School of Foreign Languages, Chengdu University of Information Technology, Chengdu, 610225 China

**Keywords:** Equity and efficiency integrated framework, Multilevel healthcare, GTL-2SFCA, Accessibility, Hubei

## Abstract

**Background:**

Equity of healthcare spatial access is essential for the health outcomes of medical investments and the welfare of populations, and efficiency of medical resource allocation is important for obtaining a supply-demand equilibrium with lower cost and higher outputs with limited inputs. However, the literature that involves both equity and efficiency in its analysis of healthcare spatial allocation is rare, and the spatial accessibility of multilevel healthcare is difficult to measure by traditional methods in a large region with diversified population distribution.

**Methods:**

To assist in solving these issues, this paper aims to build an equity and efficiency integrated analytical framework by proposing a new “GTL-2SFCA” approach to analyze the spatial accessibility of multilevel healthcare; maximum and minimum floating catchments of different levels of healthcare were assigned to ensure a combination of universal search coverage and efficient hospitalization behavior simulation.

**Results:**

The analytical framework was applied and tested in Hubei, China. Almost half of the residents (47.95%) and townships (44.98%) have access to both public general hospitals (PGHs) and primary healthcare centers (PHCs) services, 36.89% of the residents enjoy only one sufficient service, either PGHs or PHCs, and the remaining residents (15.16%) are faced with the risk of lacking access to both services. The results reveal that there are core-periphery effects of multilevel healthcare throughout Hubei and isolate clusters that have adequate access in the western region. The polarization effect of higher-level healthcare and the polycentric pattern of lower-level healthcare coexist. The multilevel healthcare shortage was identified in some areas in boundary and peripheral regions.

**Conclusions:**

This study integrates equity and efficiency into the GTL-2SFCA framework, enriches the FCA series methodologies and provides a more operational solution for evaluating the access of residents in more sophisticated spatial units to each level of healthcare. By more significantly differing and quantifying the catchment area and distance decay effect, this methodology avoids overestimating or underestimating accessibility and discovers some imperceptible spatial inequities. This study has application value for researchers and decision-makers in other scenarios and regions with significant heterogeneity in medical resources and where the population has greater mobility.

## Introduction

The trade-off between equity and efficiency is the central issue of healthcare spatial distribution. In terms of equity, the WHO initiated a strategy for universal coverage of healthcare. To achieve this goal, the Hierarchical Diagnosis and Treatment (HTD) reform has been implemented in China. This reform aims to reallocate healthcare resources among different levels and regions to meet residents’ various health needs. In terms of efficiency, healthcare allocation is always conducted under limited resource constraints; therefore, it is important to understand how to reconstruct the structure of the multilevel healthcare system and to optimize spatial distribution of medical resources to obtain a supply-demand equilibrium with lower cost and higher input-output efficiency.

Spatial equity of healthcare is beneficial for enhancing health outcomes, especially for marginal and vulnerable people. Thus, it has been analyzed in many studies ranging from healthcare professionals to healthcare institutions. Analytical methods have been developed from simple economic indices to complex spatial data models, such as from Lorenz curves, Gini coefficients [1, 2] to hot-spot analysis [3], dynamic convergence models [4] and modified 2SFCA methods [5]. Meanwhile, from the perspective of limited recourse constraints, the efficiency of healthcare has also received attention from scholars. Frontier efficiency measurement was applied to evaluate healthcare efficiency [6] and spatial location was considered a major factor influencing the input-output efficiency of healthcare resources [7]. Scholars have developed location-allocation models to analyze the healthcare access efficiencies in a limited-resource setting [8]. Spatial dependence and spatial spillover effects were considered when discussing how to select optimal healthcare centers and how to modify health policies to enhance spatial allocation efficiencies [9, 10]. However, since the equity and efficiency of healthcare allocations have been recognized as a paradox, literature that discusses both remains scarce.

Floating catchment area (FCA) series methodologies are the mainstream model used to assess the geographic accessibility to healthcare [11, 12]. The basic model has undergone various enhancements [13], including the incorporation of variable catchment sizes, age-adjusted healthcare demand, multitransportation modes, distance decay effects, and pairwise comparisons between each supply and demand [11, 12, 14–18].

From the perspective of supply, previous studies have considered accessibility to different levels of healthcare services, ranging from large comprehensive medical spots to primary healthcare services [19–22]. Recent studies have focused on the measurement of hierarchical healthcare accessibility [23–25], whereby FCA series methodologies have gradually been embedded in the framework of multilevel healthcare accessibility measurement [24, 26]. Meanwhile, scholars have considered the number of doctors and hospital beds to evaluation the capacities of different medical spots respectively [27], and different weights have been assigned to different levels of institutions to evaluate the impact of service capacities on the spatial distribution of healthcare services [28, 29]. However, medical equipment has not received enough attention, and the influence of the service capacity on the weight of the search distance within the same level has not been sufficiently analyzed, thus the preference efficiency of medical spot with better resources by the residents would be ignored.

On the demand side, population distribution and its spatial disparities have been considered the most essential factors in calculating the accessibility of healthcare. A ZIP code or a census tract has been used as a geographic unit to evaluate population needs [30], and different segments of population have been dissected to depict the heterogeneity of the population distribution [5]. However, in the existing literature, geographic units with low accuracy usually led to the overestimation of accessibility, thereby concealing local spatial inequities. Therefore, a more sophisticated spatial analysis of population patterns is called for to simulate more realistic scenarios of healthcare demand [23, 25].

In terms of the spatial barrier between supply and demand, the distance decay effect has been considered [31], while the accessible and nonassessable ranges were divided strictly in an unsmooth and nondynamic way [25]. In fact, with different service capabilities, hospitalization barriers should be defined differently. The higher the service capacity, the greater the tolerance of the residents to their hospitalizing distance; thus, the search distance of the FCA method should vary continuously. Nevertheless, literature that considers continuous decay effects is rare. Meanwhile, classic 2SFCA models have not taken the universal coverage of multilevel healthcare in a large region and extreme long-distance healthcare seeking behavior into account because of the limitation of the inefficient repetitive computing. Moreover, different modes of transportation have gained the attention of many scholars, but the estimation of hospitalization time based on assumed speeds would still bias accessibility, especially in densely populated areas [32]. How to integrate real-time travel distances into 2SFCA algorithm models and calculate the corresponding hospitalizing weight in further experimental studies is still in process [23].

**Fig. 1:**
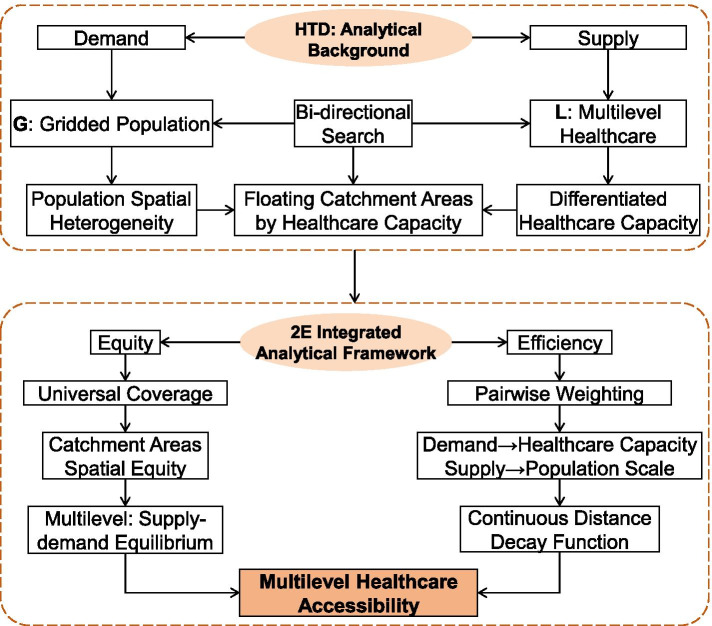
2E Integrated Analytical Framework-based GTL-2SFCA

Therefore, this paper aims to build an equity and efficiency (2E) integrated analytical framework through a new spatial accessibility assessment approach. Based on our former research on a “2R-GTL” method and the widely applied 2SFCA method [23], we presented a new “GTL-2SFCA” approach to analyze the spatial accessibility of multilevel healthcare in Hubei Province in China as a case study (Fig. 1). Considering both equity and efficiency, we suggested a universal coverage algorithm of the floating catchment areas. In addition, we created a pairwise weighting of search distances between supply and demand spots with continuous distance decay. Furthermore, we incorporated all these improved algorithms into the GTL-2SFCA assessment approach.

We select Hubei as a demonstration of our work because the population and healthcare institution distribution and topography are as diverse as those of the country. Moreover, the provincial capital of *Wuhan* and its surrounding areas experienced the serious COVID-19 epidemic, and it is of great significance to analyze the spatial distribution of healthcare to improve the public health emergency management system in this region. Furthermore, we confirm that our work can be extended to other regions or developing countries as well as expanded in scope to incorporate different public service fields by showcasing the applicability of the approach in this study.

## Methods

### Improvement of 2SFCA

Based on the 2E integrated analytical framework, this paper improves on the classic 2SFCA in the following four aspects:

1. Healthcare universal coverage. This indicates that the service scope of all medical spots in the study area can cover the whole research region, and all residential areas can access at least one medical institution at each level. The service scope of each medical spot should be directly linked to its service capability. The stronger the service capacity, the larger the scope of service, that is, the service scope of medical spots with the largest scale and best service should cover the entire study region, which is more applicable for long-distance hospitalizations in remote rural areas.
2. Continuous distance decay. In hospitalization behavior, the attenuation of the distance between medical spot and residential spot should be continuous, smooth and dynamic, that is, the longer the distance, the smaller the weight. Meanwhile, the influence of travel distance on hospitalization weight should cover the whole service range, with a varied distance decay function of different areas.
3. Pairwise weighting of searching distances. In the existing literature, 2SFCA only weighted 1st step search to distinguish the attenuation of the impact of medical spots on residents with different distances, while ignoring the residents’ selectivity of medical sites in 2nd step search [2, 26]. In this study, we weighted the two step searches in 2SFCA to highlight the multiple effects of the service capacity of medical spots and travel distance on hospitalization behavior.
4. The comprehensiveness of healthcare service capacity. Instead of only using the number of physicians or sickbeds to represent the service ability of medical spots, this study employs the number of physicians, sickbeds and specific equipment to measure the service capacity and scope of medical spots in a more comprehensive way.

### 2R-GTL

We presented a “2R-GTL” framework to assess the spatial accessibility of multilevel healthcare resources from certain spot in regions. The term “G” refers to kilometer grids, which are both the units of spatialization of the residential population and the starting points of the spatial accessibility calculation; the term “L” represents the levels of medical spots in a multilevel healthcare system according to China’s HDT reform; and the term “2R” refers to real-time traffic distance and residents’ behavioral preferences for different levels of healthcare. The spatial accessibility of each level of healthcare was distinguished and overlapped. Relevant detailed information can be found in the literature published in 2019 [23]. In this paper, the gridded population is regarded as the demander while public general hospitals (PGHs) (secondary and tertiary hospitals) and primary healthcare centers (PHCs) are taken as suppliers in the accessibility measurement, to calculate the accessibility of multilevel healthcare services in Hubei Province.

### GTL-2SFCA

**Fig. 2:**
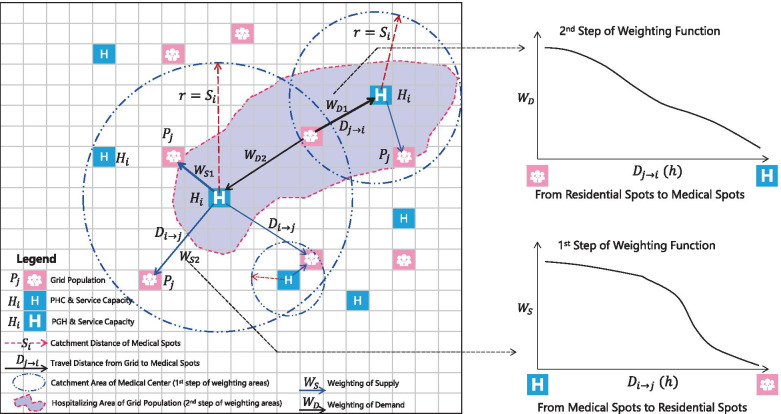
The Algorithm Overview of GTL-2SFCA

Combining the improved 2SFCA with the 2R-GTL framework, the GTL-2SFCA algorithm constructed in this paper is as follows (Fig. 2): (1) The supply and demand spots of healthcare services are positioned to distinguish PHCs and PGHs, and discretize the population data of administrative regions into gridded population data, and render the center point of the grid as the demander spots of healthcare services; (2) The service scope of each medical spot is calculated around which the search for residential spots within the service range is conducted. The real-time travel distance from the residential spot to the medical spot is calculated. Based on the travel distance, the weight (*W*_*S*_) of the medical spot (supply spot) is constructed. Then the gridded population within the service scope is in weighted to sum and the ratio of service capacity to population is calculated; (3) With the residential spot as the starting point, the real-time traveling distance between the residential spot and the medical spot that covers it in the service scope is calculated, and the weight (*W*_*D*_) of each residential spot (demand spot) is constructed. Then, the ratio of the service capacity of each medical spot to the population is weighted to sum and to obtain the healthcare accessibility index of the grid. The accessibility of PGHs and PHCs are calculated by the above steps.

#### The healthcare capacity of medical centers

The three major factors that influence healthcare capacity are beds, healthcare professionals and equipment of medical spots [33], but in the evaluation of medical resources, the importance of each factor has not been fully compared [9], and by default, they are given the same weight. However, considering the universality of medical service scenarios, the importance of health professionals needs to be emphasized [26, 34]. Therefore, to comprehensively characterize the service capabilities of the medical spots, the healthcare service capacity index is composed of the weighted sums of beds, health professionals and equipment above 10,000 *yuan*. To highlight the importance of health professionals, the weights of beds, personnel and equipment are set to 0.3, 0.4 and 0.3, respectively. Thus, the equation for the calculation of the healthcare service capacity index is as follows:

1 $${H}_i={B}_i\times 0.3+{D}_i\times 0.4+{F}_i\times 0.3$$

where, *H*_*i*_ is the service capacity index of the *i*th healthcare spot, and *B*_*i*_, *D*_*i*_, *F*_*i*_ represent the corresponding numbers of beds, health professionals and equipment above 10,000 *yuan* in the *i*th healthcare spot, respectively.

#### Measurement of catchment area

The study determines the search scope of healthcare service accessibility measurement by the principles of equity and efficiency. In terms of equity, the maximum search distance for a PGH is determined by the maximum distance from center of the area with the richest medical resources to the area’s boundary (that is, the PGH with largest service capacity in the study area must serve all residents in that area).

The maximum search distance of a PHC must not span the prefecture-level area, and it should be determined by half of the distance from the center to the border in the largest prefecture-level area. For a balance between equity and efficiency, overlapping search scopes should be avoided as much as possible to reduce unnecessary repeated calculations, and universal coverage of the study area by the search scope must be ensured.

In this study, the minimum search distance was determined by the spatial distribution of medical spots. The specific method is to calculate the maximum distance among medical spots, with half of the maximum taken as the minimum search distance of each medical spot. To make the calculation more efficient, the search scope with the smallest capacity is the minimum search distance while the search scope with the largest capacity is the maximum search distance, and the search distance from the other medical spots is determined by their service capacity. All the above methods ensure a continuous and dynamic catchment search.

Therefore, the search distance formula of each medical spot is as follows:

2 $${\displaystyle \begin{array}{c}{S}_i={dis}_{min}+\frac{\left({dis}_{max}-{dis}_{min}\right)}{\left({H}_{max}-{H}_{min}\right)}\times \left({H}_i-{H}_{min}\right)\\ {}{dis}_{min}=\frac{1}{2}\underset{i}{\max}\left({H}_1^i\to {H}_{1\cdots}\right)\end{array}}$$

where, *S*_*i*_ is the search distance (geographical distance) of the *i*th healthcare spot, *dis*_*min*_, *dis*_*max*_ are the minimum and maximum search distances of the medical spot at this level, and *H*_*max*_, *H*_*min*_ are the maximum and minimum values of the service capacity index of healthcare spots at this level, respectively, and ${H}_1^i\to {H}_{1\cdots }$ represents the distance between each medical spot and the other medical spots (within the same level).

#### Calculation of 2SFCA

The 1st step search. Given the search distance of medical spots, what follows is the calculation of the ratio of the healthcare service capacity index to the residential populations in the search scope of each medical spot (*R*_*i*_). To achieve continuous distance decay between medical spot and residential spot, time accessibility is proposed to calculate the *W*_*S*_ of residential spot. In other words, the *W*_*S*_ in each residential spot is assessed according to the traveling time distance (driving travel) from the residential spot to the medical spot within the search scope. The closer the residential spot is to the medical spot, the greater the *W*_*S*_.

After *W*_*S*_, the permanent population of each residential spot within the search scope is weighted to sum to finally obtain the ratio of healthcare service capacity to the size of the populations. The formula is as follows:

3 $${R}_i={H}_i/\sum_{j\in \left\{{S}_{i\to j}\le {S}_i\right\}}{P}_j\times \frac{1/\frac{D_{i\to j}}{\sum_{j\in \left\{{S}_{i\to j}\le {S}_i\right\}}{D}_{i\to j}}}{\sum_{j\in \left\{{S}_{i\to j}\le {S}_i\right\}}\left(1/\frac{D_{i\to j}}{\sum_{j\in \left\{{S}_{i\to j}\le {S}_i\right\}}{D}_{i\to j}}\right)}$$

where, *R*_*i*_ is the ratio of the service capacity index of the *i*th medical spot to the number of people within the search scope. *P*_*j*_ is the population of the *j*th residential spot (grid) in the search scope, *D*_*i* → *j*_ represents the traveling time from the *i*th medical spot to the *j* residential spots, and *S*_*i* → *j*_ represents the geographical distance from the *i*th medical spot to the *j*th residential spot (grid). *j* ∈ {*S*_*j* → *i*_ ≤ *S*_*i*_} represents the residential spots within the search scope of the *i*th medical spot.

After the calculation of the 1st step search, the 2nd step search is conducted from the residential spot. That is to count the medical spots covered within the catchment area of the residential spot and calculate the weighting sum of *R*_*i*_ of each medical spot as the healthcare accessibility of the residential spot. In the calculation, it is still necessary to reflect the continuous distance decay and the pairwise selectivity of health seeking behavior. In other words, residents will give priority to visiting medical spots with shorter traveling times and larger scale capacities. The shorter the distance, the greater the *W*_*D*_. The calculation formula is as follows:

4 $${A}_j=\sum_{i\in \left\{{S}_{j\to i}\le {S}_i\right\}}{R}_i\times \frac{1/\frac{D_{j\to i}}{\sum_{i\in \left\{{S}_{j\to i}\le {S}_i\right\}}{D}_{j\to i}}}{\sum_{i\in \left\{{S}_{j\to i}\le {S}_i\right\}}\left(1/\frac{D_{j\to i}}{\sum_{i\in \left\{{S}_{j\to i}\le {S}_i\right\}}{D}_{j\to i}}\right)}$$

where, *A*_*j*_ is the healthcare accessibility index of the *j*th residential spot(grid), and *i* ∈ {*S*_*j* → *i*_ ≤ *S*_*i*_} represents the medical spots with the *j*th residential spot covered within the search scope.

### Case study area and data sources

**Fig. 3:**
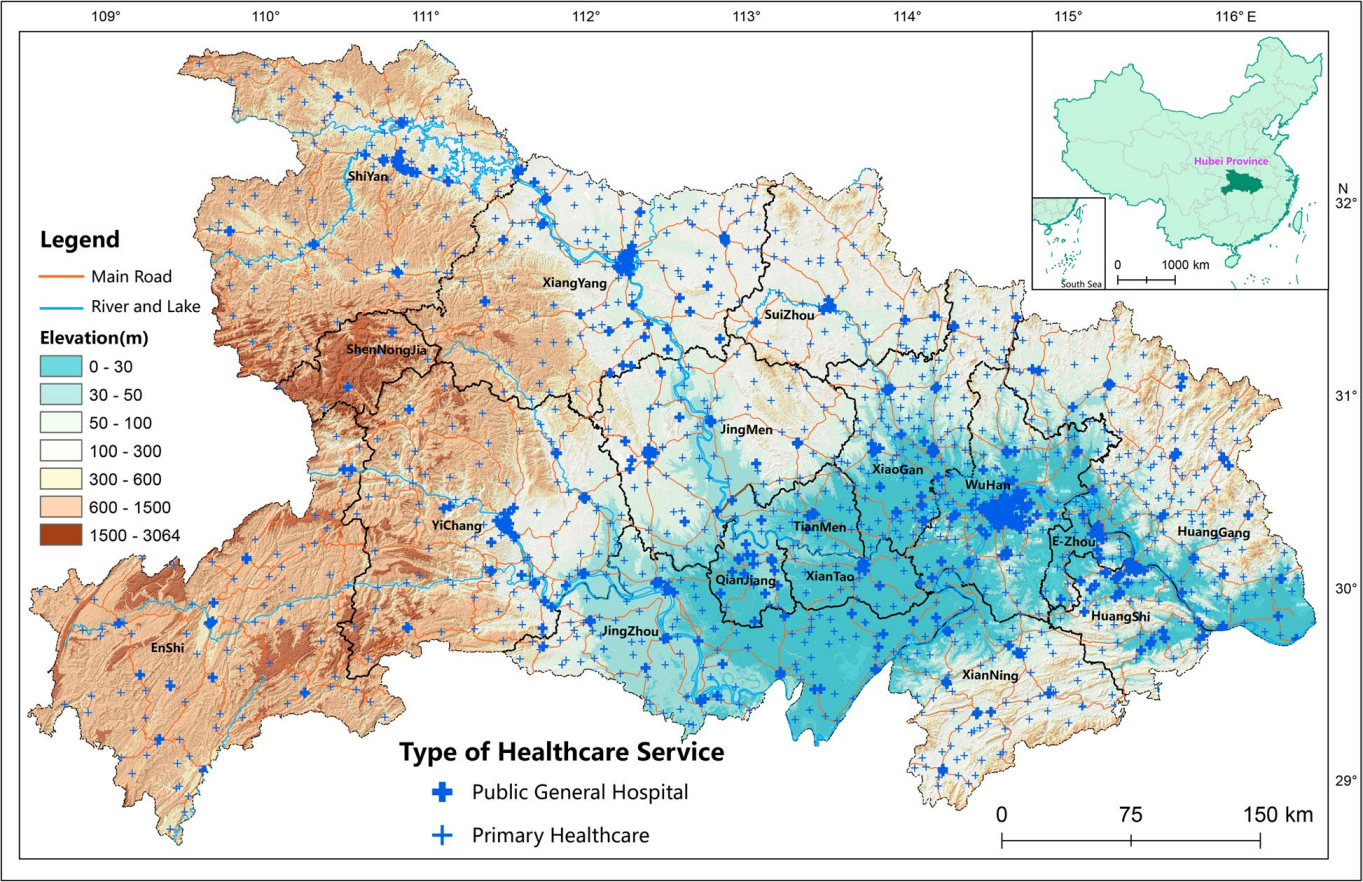
Overview of Topography and Distribution of Medical Spots in Hubei

Hubei is a province located in central China. In 2018, the province’s GDP reached 3936.66 billion yuan, with a permanent residential population of 59.17 million. The per capita GDP was 66,531.27 yuan, and the urbanization rate reached 60.30%. By 2018, there were 583 general hospitals in Hubei, including 386 PGHs. There are 1241 PHCs, including 1178 township healthcare centers, 53 community health service centers and 29 outpatient institutions. There were 393.50 thousand beds in medical spots in the whole province. According to the province’s permanent population, the number of beds in PHCs per thousand people was 6.65 in urban and rural areas, and only 5.05 in rural areas. There were 1.86 beds per thousand of rural people in township healthcare centers. There were 411 thousand healthcare professionals in Hubei, 6.9 professionals per thousand people, slightly higher than the national average of 6.8 in the same period. The number of healthcare workers per thousand population in urban areas (10.2 persons) is approximately twice that in rural areas (4.9 persons). Hubei is surrounded by mountains in the east, west and north with the *Jianghan* Plain in the central south. The mountainous area accounts for 56% of its total area, the hilly area accounts for 24% and the plain area accounts for 20% (Fig. 3). There is obvious population spatial heterogeneity in Hubei. High quality medical resources are mainly distributed in urban areas and densely populated areas. There is a significant disparity in medical services between urban and rural areas, plain and mountainous areas, eastern and western areas, and between PHCs and PGHs.

The data used in the study mainly include healthcare data, population data and night light images. The healthcare data are mainly the detailed attributes of various types of medical and healthcare spots in Hubei. The main attribute fields include institution name, address, number of beds, number of health professionals, and number of pieces of equipment valuated at over 10,000 *yuan*. The healthcare data are from the official statistics of the Health Commission of Hubei Province (2018) (http://wjw.hubei.gov.cn/). Meanwhile, based on the Baidu geocoding API (http://api.map.baidu.com/geocoding/v3/), the address of medical spots is converted into longitude and latitude to prepare the spatial data set of medical spots in Hubei in 2018. The population data comes from the statistical yearbook of all prefecture-level cities in Hubei and the *China Statistical Yearbook (Township volume)*, forming the spatial population dataset of 1222 township units. The night light image is from NOAA’s NPP/VIIRS night light dataset (https://ngdc.noaa.gov/eog/viirs/index.html).

## Results

### Gridded population and multilevel healthcare capacity

#### Gridded permanent residential population

**Fig. 4:**
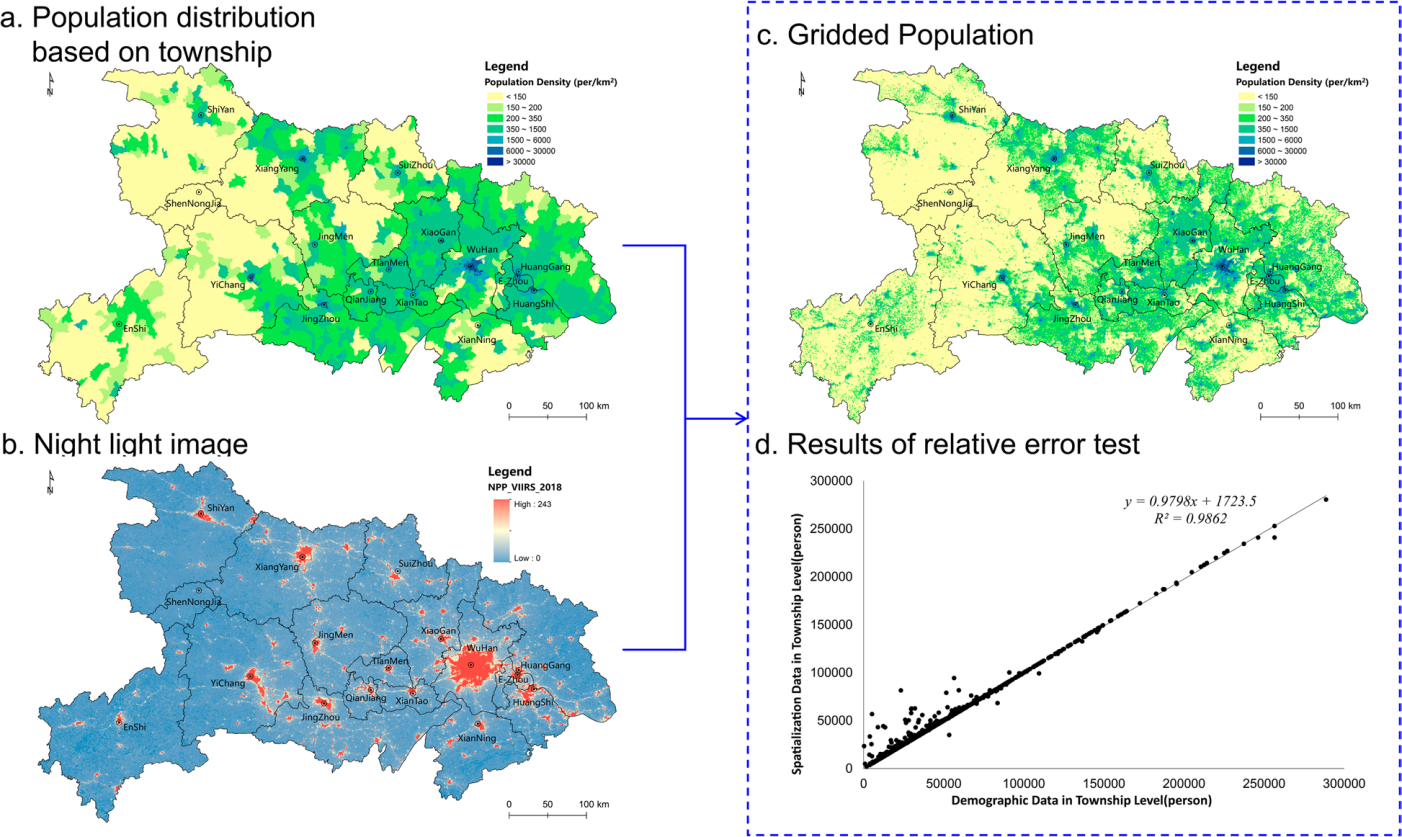
Processing of Population Gridding in Hubei

In the population distribution figure based on township statistical data in Hubei (Fig. 4), the population density significantly corresponds to the topography; that is, most of the population is concentrated in the *Jianghan* Urban Plain Agglomeration comprising *Wuhan*, with *Jingmen*, *Xiangyang* and *Shiyan* in the *Hanjiang* Basin as densely populated areas. In contrast, the population density of most other areas is less than 150 people per km^2^. Overall, Hubei is more densely populated in the east and south than in the west and north, with a conspicuous tendency toward densely populated urban areas. Based on the nighttime light image, we obtained the spatial distribution dataset of the gridded population. The correlative analysis of the gridded population data and the statistical population data at the township level revealed a very close linear relationship (Fig. 4), and the regression fitting degree *R*^*2*^ was 0.9862. The gridded population of most townships is quite close to the statistical population, which shows that the gridded population data bears a true depiction of the high-resolution spatial distribution pattern of the population, as well as excellent fundamental data for the analysis of healthcare spatial accessibility.

The spatial distribution of the gridded population shows more details of the spatial population differentiation in local areas. At the grid-scale, there is a more obvious trend in which the population of Hubei is more densely distributed along the *Yangtze* River and *Hanjiang* River, mainly in urban areas and particularly in *Wuhan*. However, there are some densely populated areas in the western mountainous areas, such as *Enshi* and the southern of *Shiyan*. It should be noted that the spatial distribution of the population in Hubei poses many challenges to the equity of healthcare accessibility. It is significant to meet the diversified demands of many people for multi-level healthcare in densely populated urban areas. Simultaneously, it is equally crucial to provide available and accessible healthcare to people living in remote mountainous areas.

#### Differentiated multilevel healthcare capacity

**Fig. 5:**
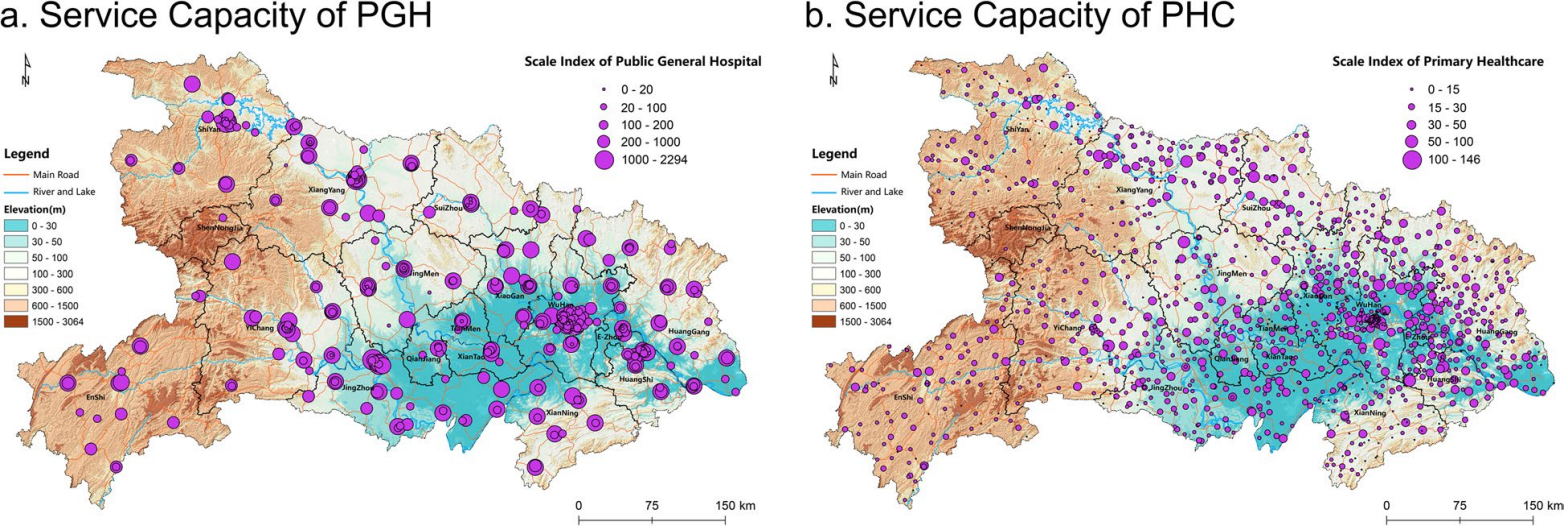
Spatial Distribution of PGH and PHC Service Capacity in Hubei

Based on the calculation of the healthcare capacity index, Fig. 5 shows the spatial distribution pattern of the PGH and PHC capacity in Hubei. The number and capacity of PGHs in *Wuhan* rank first in Hubei. In 2018, there were 91 PGHs in *Wuhan*, accounting for 23.58% of the whole province, while *Shennongjia* had only one PGH. In terms of the service capacity, there is a significant provincial internal differentiation in which the provincial average service capacity index of PGHs is 196.92 while the SD is 259.78. According to the average value of each prefecture’s PGH service capacity index, the highest is *Wuhan*, reaching 263.34, while *Shennongjia* is only 82.80.

The average value of the PHC service capacity index was 30.12 with an SD of 19.80. Compared with the PGH service, the PHC service capacity and the province internal difference are smaller. The PHCs still cluster in the southeast plain areas and are sparse in the western mountainous areas, where the service capacity is small. In terms of spatial disparity, *Wuhan* has the largest number of PHCs, reaching 142 with only 7 in *Shennongjia*. In terms of capacity, *Qianjiang* has the largest average value of PHC service capacity, reaching 46.97; *Shennongjia* has the smallest average PHC capacity, only 8.44, with the other prefecture cities’ PHC service capacity all above 20. This indicates that there is comparatively smaller difference in the PHC service capacity index among prefectures except for *Shennongjia*.

Spatial autocorrelation analysis was carried out on the spatial distribution of PGH and PHC service capacity. The results showed that the Moran*’s I* index of PGH and PHC service capacity was − 0.0067 (*Z*: − 0.12, *p*: 0.91) and 0.12 (*Z*: 8.03, *p*: 0.00), respectively. This indicates that there is no obvious spatial agglomeration or decentralization trend in the spatial distribution of PGH service capacity, while PHC service has a significant trend of local agglomeration. According to Fig. 5, the PHC service capacity in southeastern Hubei is remarkably higher than that in the western mountainous area, creating a significant spatial disparity. In contrast, PGH service capacity is more equal in terms of spatial distribution than that of PHCs, which reveals that the major factor shaping the spatial differentiation of multilevel healthcare services in Hubei is PHCs. The spatial differentiation of PHC services is greater than that of PGH services between urban and rural areas, mountainous and plain areas, and between eastern and western areas. The poverty and inadequacy of PHC services have become major causes of the inequity of healthcare services in rural and mountainous areas.

### Multilevel healthcare spatial accessibility based GTL-2SFCA

#### Differentiated hospitalizing time

The average traveling time from residential spots to each medical spot in the catchment area was deemed as hospitalizing time per grid to characterize the medical traffic convenience. As shown in Table 1, residents in more than 80% of the areas need to travel for more than 1 *h* for PGH service, while those in more than 25% of the areas spend more than 2 *h*. In terms of the proportion of the population covered by the corresponding time, 42.44% of permanent residents can access to PGH service within 1 *h*, with 7.82% requiring more than 2 *h*. Most of the residents (84.41%) can access PGH service within 1.5 *h*. In terms of PHCs, only 0.22% of the total area of Hubei accesses PHC service within 0.2 *h*, while more than 75% within 0.2 ~ 0.6 *h*, and 2.85% require more than 1 *h* to access service. It shows that most areas can access a PHC in approximately 0.5 *h*, but the hospitalizing time of local areas is far above the average level of all areas. In terms of the population proportion covered by the corresponding time, only 0.23% of the residents access a PHC in less than 0.2 *h*, and only 0.45% more than 1 *h*. More than 93% of the population can access a PHC within 0.2 ~ 0.6 *h*, which reveals that most of the population is covered in the 0.5 *h* service circle of PHCs.

**Table 1:** The Proportion of Area and Population of Hospitalizing Time Zone

Healthcare Level	Hospitalizing Time Zone (*h*)	Grid number	Area Proportion	Population(million)	Population Proportion
PGH	< 0.5	184	0.87%	7.58	12.92%
	0.5 ~ 1	3040	14.31%	17.31	29.52%
	1 ~ 1.5	8675	40.84%	24.61	41.97%
	1.5 ~ 2	3875	18.24%	4.56	7.78%
	2 ~ 3	4582	21.57%	4.20	7.16%
	> 3	886	4.17%	0.39	0.66%
PHC	< 0.2	46	0.22%	0.14	0.23%
	0.2 ~ 0.4	4603	21.67%	28.16	48.01%
	0.4 ~ 0.6	11,619	54.70%	26.56	45.29%
	0.6 ~ 0.8	3364	15.84%	2.86	4.87%
	0.8 ~ 1	1005	4.73%	0.67	1.15%
	> 1	605	2.85%	0.27	0.45%

Note: The cutoffs of hospitalizing time zones were comprehensively determined by the research team’s questionnaire survey [23] and policy planning of the Chinese government [26]

#### Healthcare spatial accessibility of PGHs and PHCs

**Fig. 6:**
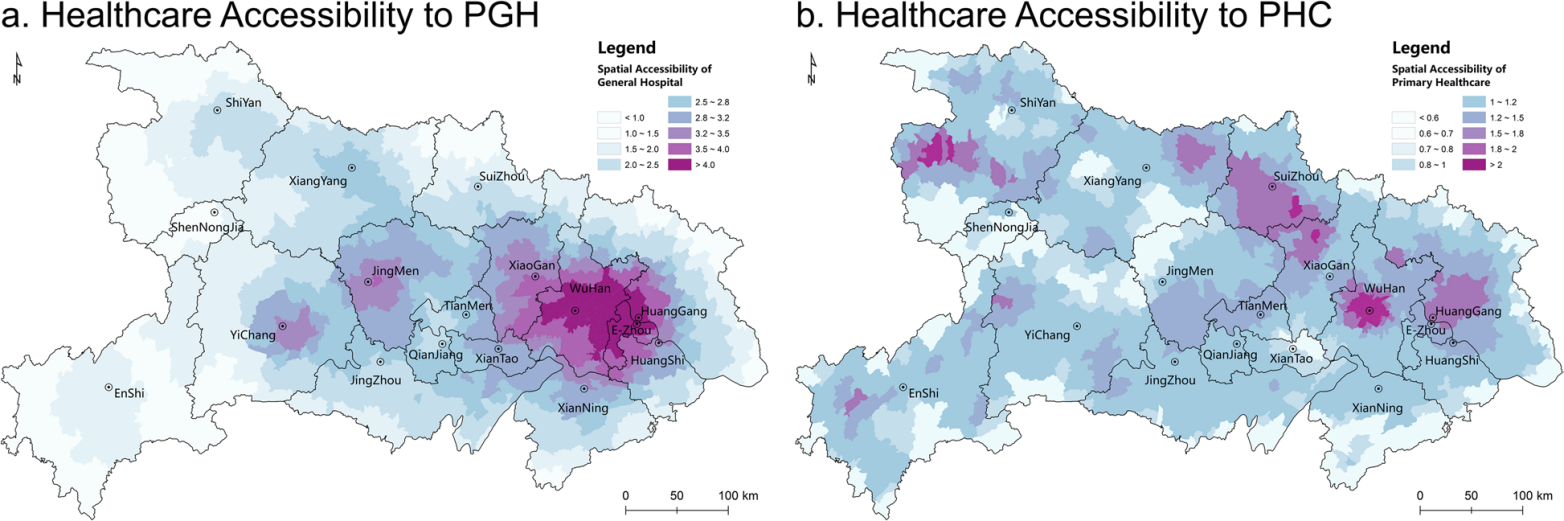
Healthcare Spatial Accessibility to PGHs and PHCs in Hubei

Based on the GTL-2SFCA method, the accessibility on the grid-scale of PGHs and PHCs is calculated and aggregated to the township scale (mean value) to measure the accessibility of the township to visit multilevel healthcare services (the accessibility index is enlarged by 1000 times for a clearer display. Therefore, the PGH accessibility index range is 0.37 ~ 4.97, and the PHC range is 0.27 ~ 3.12). Figure 6a shows the spatial pattern of accessibility to PGHs in Hubei, depicting that the *Wuhan* urban agglomeration is the core of PGH service, and that accessibility gradually decreases in the surrounding areas. The accessibility index of *Wuhan* and most of *E’zhou* and *Huanggang* is higher than 4, which decreases to below 2.5 in a concentric circle, showing that there is a high spatial agglomeration in Hubei. On the whole, the accessibility pattern of PGH features a high level of access in the east and a low level of access in the west with extremely significant spatial differentiation(Moran*’s I*: 2.53, *Z*: 195.29, *p*: 0.00). The disparity can be found not only in the eastern and western areas and between the mountain and plain areas but also between urban and rural areas. Compared with the spatial pattern of PGH service capacity (Fig. 5a), accessibility shows more significant spatial heterogeneity and aggregation, which verifies that residents’ spatial preference for healthcare seeking together with healthcare service capacity deeply shapes the spatial differentiation of healthcare accessibility. This results in greater healthcare accessibility of core areas and weakened accessibility in remote mountainous and rural areas.

Compared with PGHs, the overall spatial pattern of PHCs bears no obvious differences between the east and the west, but there are certain characteristics of north-south differentiation (Fig. 6b). Regarding the spatial distribution of high value areas of PHC accessibility, most are distributed in the northern area with no obvious characteristics of urban clustering. Regarding the spatial distribution of low-value areas, most are concentrated in the peripheral zone of the provincial boundary and the western mountainous areas. Meanwhile, many low-value areas are distributed at the junction of administrative districts, such as *Yichang*, *Jingmen* and *Xiangyang*, *Tianmen* and *Xiantao*. The spatial autocorrelation analysis of the spatial pattern of the accessibility to PHCs (Moran’s *I*: 6.77, *Z*: 523.50, *p*: 0.00) shows that compared with PGH service, accessibility to PHC services carries more significant spatial differentiation and agglomeration, indicating that PHC services are the main factor affecting the inequalization of healthcare service in local areas.

In China, the PHCs are mostly distributed in administrative centers and are closely related to the pattern of administrative divisions. As a result, the areas where there is a shortage of PHCs are largely in the boundaries of administrative. Meanwhile, it should be noted that there are also high-value areas of accessibility to PHC service in some rural areas, mainly attributing to a particular PHC capacity and an appropriate population density have brought about a lower degree of competition among residents for healthcare services. In addition, the comparatively higher population density of many urban areas, combined with the lower capacity of PHC services, intensifies residents’ competition for PHC services, consequently weakening the accessibility level of some urban areas.

#### Multilevel healthcare accessibility

**Fig. 7:**
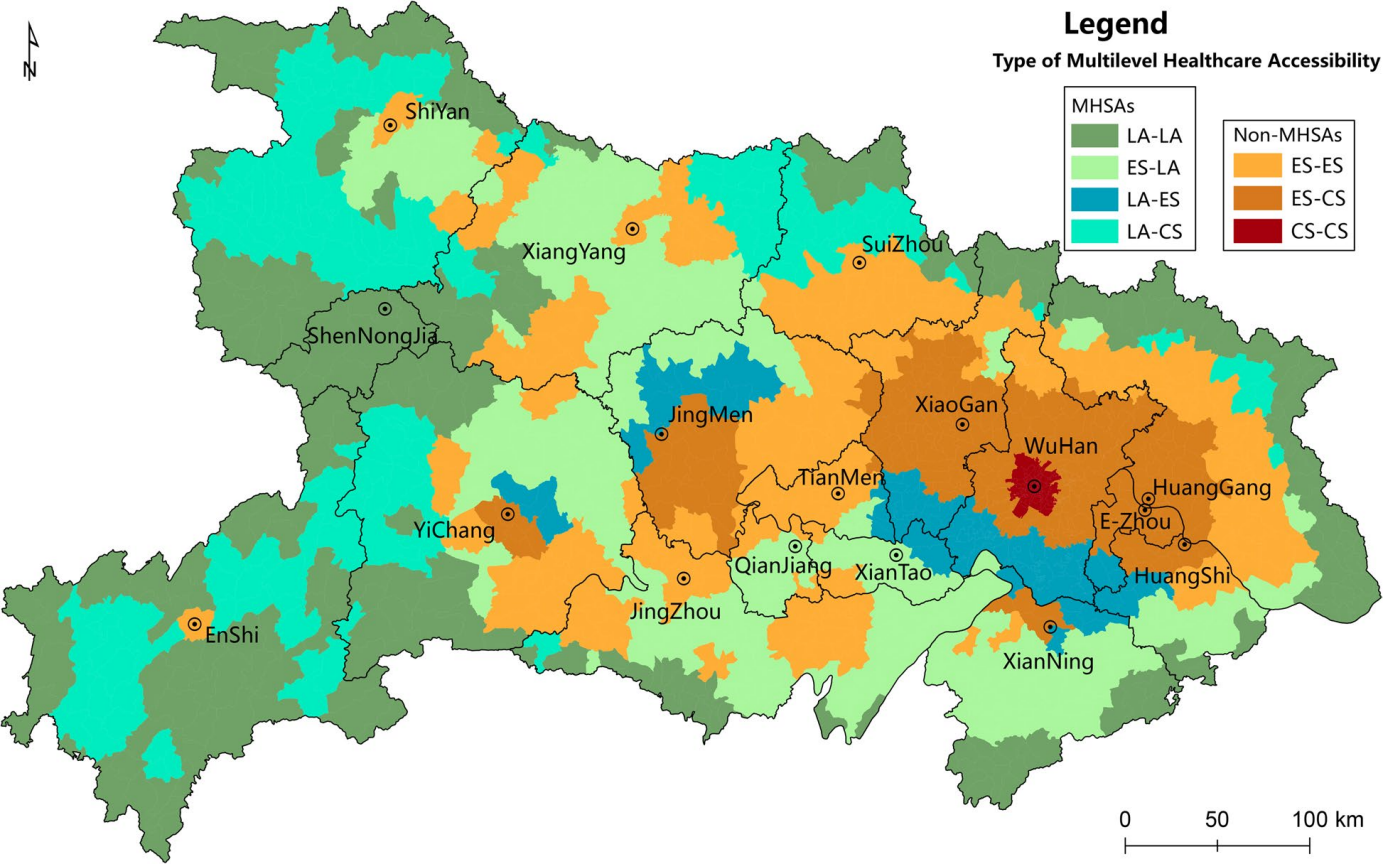
Spatial Distribution of Multilevel Healthcare Accessibility in Hubei. (**LA-LA**: Overlapping Areas of LA in PHC and PGH; **LA-ES:** Overlapping Areas of LA in PHC and ES in PGH; **LA-CS:** Overlapping Areas of LA in PHC and CS in PGH; **ES-LA:** Overlapping Areas of ES in PHC and LA in PGH; **ES-ES:** Overlapping Areas of ES in PHC and PGH; **ES-CS:** Overlapping Areas of ES in PHC and CS in PGH; **CS-CS:** Overlapping Areas of CS in PHC and PGH)

The spatial distribution of multilevel healthcare accessibility in Hubei is comprehensively displayed by an overlapping analysis of the spatial patterns of PGH and PHC accessibility (Fig. 7). Referring to the existing classification [23, 26] and frequency distribution of accessibility indices, this paper assigns 2, 3 and 1, 2 as the cutoffs of PGH and PHC accessibility indices and classifies the province into three types of areas: Lack Areas (LA), Effective Service Areas (ES) and Core Service Areas (CS) to provide a characterization of multilevel healthcare accessibility and to recognize the multilevel healthcare shortage areas (MHSAs).

Overall, the spatial pattern of multilevel healthcare accessibility in Hubei has strong differentiation and various accessibility types are staggered by area. The healthcare accessibility of the *Wuhan* urban agglomeration is distributed in a central circle. The degree of accessibility gradually decreases from the central area to the outskirts and surrounding cities, with a greater decline rate of PHCs than of PGHs. In the western of Hubei, the multilevel healthcare shortage areas and non-shortage areas are staggered. The areas with both PGH and PHC accessibility in core areas are only located in the central area of *Wuhan*, while the peripheral areas demonstrate an overlap of effective areas of PHCs and core areas of PGHs (ES-CS); there are some even more peripheral areas in the effective areas of both PHCs and PGHs (ES-ES). ES-ES areas are not only distributed in the periphery of ES-CS areas but are also sporadically distributed in the urban areas of western prefectures. The areas located in the PHC effective areas but that lack PGHs (ES-LA) are widely distributed in the western and northern mountainous areas, such as *Enshi*, *Shiyan* and *Suizhou*. The areas lacking PHC but in the service core of PGHs (LA-CS) are mainly distributed on the southern edge of the *Wuhan* metropolitan. Meanwhile, there are also LA-CS areas on the urban fringes of *Jingmen* and *Yichang*. This shows that the resident on the urban fringe is prone to the risk of insufficient PHCs, even though they have easy access to PGHs. Similarly, the areas lacking PHCs and ineffective areas of PGHs (LA-ES) are widely distributed on the periphery of LA-CS areas, further revealing that many residents in urban or suburban areas are facing shortages of PHC services despite their effective access to PGHs. The areas lacking both PHCs and PGHs (LA-LA) are the areas with the worst accessibility to healthcare services, mainly distributed along the administrative boundary of the province and widely found in western remote mountainous areas, such as *Shennongjia*, *Enshi* and *Shiyan*.

According to the statistics for townships and population (Table 2), we found that ES-ES areas bear the largest number of townships, corresponding population and the highest population proportion. Whereas LA-ES areas witness the smallest and the lowest values of the above 3 indices. Obviously, the township proportion and population proportion correspond in various types of areas. Overall, 47.95% of the residents have access to PHC and PGH services, accounting for 44.98% of all township units. Meanwhile, 12.46% of the residents have access to PHC services, but lacked PGH services. In contrast, 24.43% of the residents have access to PGH services, but they face the risk of a shortage of PHC services. In addition, 222 (18.39%) township units and 8.72 million (15.16%) people face the risk of a lack of both PHC and PGH services. The long distance of healthcare visits due to living in a remote location in mountainous rural areas, together with insufficient healthcare institutions at both the PHC and PGH levels in LA-LA type areas, led to the worst healthcare accessibility outcomes for the local residents.

**Table 2:** The Area, Population and Their Proportion of Corresponding Type Areas of Multilevel Healthcare Accessibility

Type Areas	PHC Accessibility Type	PGH Accessibility Type	Township Number	Township Proportion	Population (million)	Population Proportion
LA-LA	LA	LA	222	18.39%	8.72	15.16%
LA-ES	LA	ES	227	18.81%	10.47	18.22%
LA-CS	LA	CS	68	5.63%	3.57	6.21%
ES-LA	ES	LA	147	12.18%	7.16	12.46%
ES-ES	ES	ES	235	19.47%	11.20	19.49%
ES-CS	ES	CS	219	18.14%	11.19	19.47%
CS-CS	CS	CS	89	7.37%	5.17	8.99%

## Discussion and conclusions

This study built an equity and efficiency integrated analytical framework by proposing a new “GTL-2SFCA” approach to analyze the spatial accessibility of multilevel healthcare The results of the case study of 2E integrated GTL-2SFCA in Hubei shows that almost half of the residents (47.95%) and townships (44.98%) are accessible to public general hospital (PGH) and primary healthcare centers (PHC) service at the same time, and 36.89% of the residents can only enjoy one sufficient service between PGH and PHC, while the remaining residents (15.16%) are faced with the risk of dual lacking.

The innovation of this study is mainly reflected in the following three aspects: (1) This study integrates equity and efficiency into a framework of multilevel healthcare spatial accessibility analysis. Maximum and minimum floating catchments of different levels of healthcare were assigned to ensure a combination of universal coverage search and efficient hospitalizing behaviors simulation in a large region. (2) The study proposed a 2R-GTL approach to include more spatially disaggregated population distribution, real-time travel distance, and residents’ multilevel healthcare seeking spatial preferences into 2SFCA methodology. (3) The study provided a continuous distance decay method with pairwise weights between supply spots and demands spots taking into account the healthcare service capacity and residents’ hospitalizing spatial preferences. The weights were assigned continuously, dynamically, and they are different between the 1st and the 2nd step in the 2SFCA, and among each residential and medical spot.

The findings of this paper have certain strengths compared with the existing literature. First, the results of the case study reinforce the finding of McGrail and Humphreys that there are core-periphery effects of healthcare [5], however, we also found that there are isolated clusters that have adequate access to multilevel healthcare in the western region in the peripheral areas in Hubei. Second, the findings for multilevel healthcare are in line with those of Qin et al., who found that the polarization effect of higher-level healthcare (PGH) and the polycentric pattern of lower-level healthcare (PHC) coexist [4]. Furthermore, the finding that the multilevel healthcare shortage areas were designated in some boundary and edged regions took this research field a step forward. This phenomenon indicates that under the government-led public healthcare allocation system by administrative division, the fringe effect is the major reason for the inequity of healthcare. Third, recent research has paid attention to the spatial equity and efficiency of healthcare allocation [2, 34, 35], this methodologically oriented study aims to address how to integrate equity and efficiency into the evaluation of healthcare accessibility. By considering universal coverage for all residents and the efficiency of the nearest medical treatment, the range of values of the accessibility index and its variability among regions is smaller in this study than those of similar studies [24, 26]. This means that our methodology avoids overestimating accessibility in local high-density medical spots areas, and also avoids underestimate in remote, isolate areas [2]. In the context of population migration, cross-district medical treatment is becoming increasingly usual. On the one hand, the best medical institutions in the region can always attract remote seekers, especially those with severe diseases; on the other hand, those medical institutions are always overcrowded so that their accessibility for neighboring residents is lower due to the crowding out effect. The methodology in this study can better evaluate the impacts of these cross-district medical treatment behaviors on accessibility. Because of this, 2E integrated GTL-2SFCA shows robust and reliable adaptability of accessibility evaluation when the medical resources and patients are cross-district migrating against a backdrop of hierarchical diagnosis and treatment reforms.

The findings of this study yield some valuable implications for healthcare policy. Health China 2030 mentions that until 2030, the neighborhood physical reach to the PHCs by individuals should be within 15 min (the State Council of China, 2016). Therefore, allocating more primary care resources in the PHC shortage areas is of great significance to enhance the equity of multilevel healthcare systems. To accommodate underserved populations is the crux of achieving a fair and efficient allocation in a large area with limited resources, particularly in developing countries and regions. Operational solutions include restructuring upper-level healthcare resources and reinforcing the medical consortia of PGHs and PHCs between healthcare core areas and MHSAs, and between public and private hospitals are operational solutions. From the case study in Hubei, we confirm that these findings have implementations for researchers and decision-makers for obtaining a deep understanding of the spatial equity and efficiency of healthcare resource allocation. The GTL-2SFCA approach can be applied to measure access to other public services in other scenarios and regions, especially regions with significant heterogeneity in public service resources.

Future research efforts could focus on the universal coverage of underserved areas with extremely long distances from healthcare institutions with limited healthcare resources. In areas with high heterogeneity of healthcare resources and population distribution, it is not practical to achieve absolute spatial equity of healthcare. How can residents’ traffic inconvenience be improved to access medical services? How can we monitor finer-scale population density? How can inclusive and interoperable medical security insurance systems be improved? And how can a cooperative and synergic multilevel healthcare provision system be built among different regions? All these questions are worthy of long-term and in-depth study.

## Data Availability

The datasets used and/or analyzed during the current study are available from the corresponding author on reasonable request.

## Appendix

Table 3

**Table 3:** Nomenclature table

Symbol	Descriptions
*2E*	Equity and efficiency
*2R*	Real-time traffic distance and residents’ behavioral preferences
*2SFCA*	Two steps of floating catchment area
*GTL*	Grids to levels of medical spots
*PGHs*	Public general hospitals
*PHCs*	Primary healthcare centers
*CS*	Core Service Areas
*ES*	Effective Service Areas
*LA*	Lack Areas
*MHSAs*	Multilevel healthcare shortage areas
*LA-LA*	Overlapping Areas of LA in PHC and PGH
*LA-ES*	Overlapping Areas of LA in PHC and ES in PGH
*LA-CS*	Overlapping Areas of LA in PHC and CS in PGH
*ES-LA*	Overlapping Areas of ES in PHC and LA in PGH
*ES-ES*	Overlapping Areas of ES in PHC and PGH
*ES-CS*	Overlapping Areas of ES in PHC and CS in PGH
*CS-CS*	Overlapping Areas of CS in PHC and PGH
